# Two-Dimensional Crystallization Procedure, from Protein Expression to Sample Preparation

**DOI:** 10.1155/2015/693869

**Published:** 2015-08-27

**Authors:** Qie Kuang, Pasi Purhonen, Hans Hebert

**Affiliations:** ^1^Department of Biosciences and Nutrition, Karolinska Institutet, Novum, 14183 Huddinge, Sweden; ^2^School of Technology and Health, KTH Royal Institute of Technology, Novum, 14183 Huddinge, Sweden

## Abstract

Membrane proteins play important roles for living cells. Structural studies of membrane proteins provide deeper understanding of their mechanisms and further aid in drug design. As compared to other methods, electron microscopy is uniquely suitable for analysis of a broad range of specimens, from small proteins to large complexes. Of various electron microscopic methods, electron crystallography is particularly well-suited to study membrane proteins which are reconstituted into two-dimensional crystals in lipid environments. In this review, we discuss the steps and parameters for obtaining large and well-ordered two-dimensional crystals. A general description of the principle in each step is provided since this information can also be applied to other biochemical and biophysical methods. The examples are taken from our own studies and published results with related proteins. Our purpose is to give readers a more general idea of electron crystallography and to share our experiences in obtaining suitable crystals for data collection.

## 1. Introduction

Membrane proteins are closely associated with both cell and intracellular membranes. They have diverse but important functions at the cell surface as channels, transporters, receptors, enzymes, and anchors for other proteins. Many of them are of medical interest as drug targets [1]. Although membrane proteins are abundant and form 25–30% of all proteins [2], they are still structurally less well characterized than soluble proteins.

The difficulties with structural studies of membrane proteins derive from their hydrophobic properties and close interaction with lipids. The majority of atomic structures have been determined by X-ray crystallography. Although some structures are solved using lipidic crystallization techniques [3], most of them are still studied from detergent solubilized samples, in the absence of lipids. The lipid environment is normally critical for the correct folding of membrane proteins as well as preserving their structures and functions [4, 5]. In contrast to X-ray crystallography, electron crystallography (EC) analyzes two-dimensional (2D) crystals where protein molecules are embedded in lipid environments. Thus, this method is particularly suitable for structural studies of membrane proteins and may prevent conformational artefacts that can be introduced due to the absence of lipids. Although producing large and well-ordered 2D crystals as well as well-diffracting 3D crystals is still difficult, even medium-resolution 2D crystals can provide valuable structural information.

Recent advances in single-particle reconstruction (SPR) [6], where macromolecules in solution are analyzed using electron microscopy, have led to a breakthrough in obtaining high-resolution structures of macromolecular complexes, including membrane proteins, to near atomic resolutions [7]. As compared to SPR, the benefits of EC are that protein samples can be studied within a membrane environment and it is also well-suited for proteins with low molecular weights.

Membrane proteins are seldom found in native membranes at high concentrations. Such rare cases are, for example, bacteriorhodopsin in purple membranes [8] and Na^+^, K^+^-ATPase in pig kidney [9]. Hence, overexpression is usually preferred to amplify the materials. The following purification step, which is aimed at isolation of the target protein, directly affects the final crystal quality. Usually many crystallization conditions are screened for purified samples in order to obtain large and well-ordered 2D crystals suitable for data collection.

In this review different aspects concerning overexpression, purification, crystallization, and sample preparation are discussed. Our purpose of this review is to (1) provide a general introduction of this procedure; (2) share our experience to obtain large and well-ordered 2D crystals; and (3) emphasize less discussed parameters during the process. The procedure and the general concepts can also be applied to material preparation in SPR and X-ray crystallography. Examples are taken from studies with different potassium channels [10] and members of the MAPEG (membrane-associated proteins in eicosanoid and glutathione metabolism) protein family [11].

## 2. The Experimental Procedure

### 2.1. Recombinant Expression in* Escherichia coli*

The target protein is usually recombinantly overexpressed in different host systems, for example, bacteria, yeast, insect, mammalian cells, or cell-free expression systems [12]. The bacteria approach is very robust and easy to work with as compared to other systems. Thus, it is probably always the first choice to be tested, if the expressed protein is active without posttranslational modifications. The recombinant expression in* Escherichia coli* (*E. coli*) is introduced in the following section.

**Figure 1:**
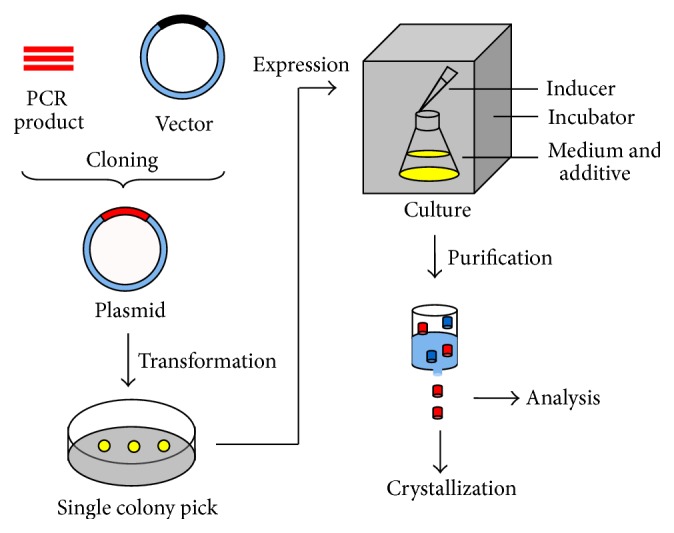
Flowchart of overexpression and purification of recombinant protein in* E. coli*. The PCR product of the target gene (red), the replaced sequence in the vector (black), the overexpressed target protein (red), and other impurities (dark blue) are depicted. The* E. coli* culture (yellow, the single colony is in yellow as well) can express the target protein after induction. Purification is performed on different columns to obtain a pure sample (red). The purified sample can be analyzed by diverse biochemical and biophysical methods further, including SPR, or it can be reconstituted into crystals.

Figure 1 shows the flowchart of overexpression of the target proteins. The target protein gene is amplified by polymerase chain reaction (PCR) and then the PCR product is ligated with the selected commercial vectors (such as the pET system from Novagen and the pBAD system from Invitrogen). The plasmids harboring the gene of interest are transformed into the competent* E. coli* cells by either chemical preparation or electroporation. Chemically prepared competent cells are more widely used and work well in most cases for penetration of the plasmids. Expression of the target proteins is usually started from the cells grown in one single colony. The culture is grown until it reaches a certain phase. Afterwards, it is induced to produce the target protein by certain chemicals depending on the choice of the plasmids and strains.

All factors, for example, choices of plasmids, strains, expression conditions, and fusion tags, affect the yield of the production, which further influence the down streamed purification and crystallization steps. Only the choice of strains and expression conditions are discussed here. A more complete discussion of the factors can be found elsewhere (e.g., [13]).

*(A) Strain*. Many different strains exist and they have their own advantages. For membrane protein, C41 and C43 (both from the BL21DE3 strain) should be considered, since these derivatives not only have a slower transcription level [14, 15], but also increase the size of the area to possibly accommodate a larger amount of target proteins [16]. The original BL21DE3 and the newly designed Lemo21DE3 strains [14] are worth testing as well.

*(B) Expression Condition*. Expression condition is another important parameter to be investigated, for example, culture media, inducer concentration, the induced time point, and the culture temperature. The terrific broth (TB) media contain more nutrition than the Luria-Bertani broth media, making the TB media more commonly used for expression of membrane proteins. Other media are also possible and may be specific for each project. The inducer depends on the plasmid and the strain. Isopropyl *β*-D-1-thiogalactopyranoside (IPTG) and its affecting pET system usually work well. In addition, the tightly regulated pBAD promoter may be considered if the leaky expression of the target protein is highly toxic to the host cells [17]. The normally added IPTG concentration is between 0.5 and 1 mM. However, there are special cases where extreme concentration is used (e.g., hMPGES1 (microsomal prostaglandin E synthase 1 from* Homo sapiens*, a MAPEG member) is induced by 3 mM IPTG [18]). The inducer concentration may be linked to the culture temperature. A high temperature (e.g., 37°C) may be combined with a higher inducer concentration as well as a shorter culture period. If the target protein yield was too low or protein aggregates were formed (protein aggregates precipitate as inclusion bodies in* E. coli*), culturing at a low temperature, for example, at 20°C overnight, may help. Other additives may be considered as well [19]. One of them, glucose, is commonly used to repress the leaky expression. The requirement of the additives may be related to the function of the target protein; for example, when KvAP (a voltage gated potassium channel from* Aeropyrum pernix*) is over-expressed, BaCl_2_ is added to block conduction of potassium ions in the protein, since the conduction may be harmful to the host cells [20, 21].

### 2.2. Protein Purification

Purification is a critical step to obtain pure samples for further studies. It separates the overexpressed target protein among other proteins (called impurities) based on the properties of the target protein. Any property can be used to distinguish it from impurities, such as affinity interaction, molecular weight, surface charge, and hydrophobic interaction [22]. Normally, this step is performed on different kinds of columns (Figure 1). The solution including both the target protein and impurities is loaded on the column, followed by washing out the impurities and then eluting and collecting the target protein. If the target protein does not bind to the column whereas impurities do, the unbound solution containing the target protein can be collected.

The most commonly used strategy in purification is to add an extra tag to the target protein. This tag can aid the protein expression and/or purification. The tags can be fused to either the N- or C-terminal end of the target protein. The efficiency may be different for different constructs. Maltose binding protein (MBP) and soluble glutathione transferase (GST) are two commonly used tags to perform the dual functions. These partners are stable proteins which may increase the target protein solubility; meanwhile, they can bind to the ligands that have been covalently linked to the columns already (called affinity purification). A newly identified tag, called Mistic (membrane-integrating sequence for translation of integral membrane protein constructs), has been suggested to aid the target protein inserting into the membrane [23, 24]. These large tags (several tens of kilodaltons) may be necessary to be erased after purification by proteases. On the other hand, small tags (such as a polyhistidine tag) exist and do not need excising. These tags usually do not influence the protein activity and can improve the purification efficiently. In fact, the structural determination of hLTC_4_S (leukotriene C_4_ synthase from* Homo sapiens*, a MAPEG member) was aided by the histidine tag [25].

Size exclusion (also called gel filtration) purification separates proteins by their different molecular weights. Notice that the protein shape can influence the retarded time in elution as well. Ion exchange purification relies on the charge interactions between the sample and the column. It has either cation or anion exchange chromatography depending on whether the positively (cation exchange) or negatively (anion exchange) charged proteins are attracted to the column. Besides the charged residues in the sample, the buffer pH affects the charge interactions. The hydrophobic interaction purification divides proteins based on their different hydrophobicity. However, it is probably less used for membrane proteins.

Centrifugation can work as a crude purification tool as well. Since different cell organelles have different sizes and densities, they can be separated at different centrifugal forces. For membrane proteins, centrifugation and ultracentrifugation are general procedures to remove cell debris (including inclusion bodies) and soluble protein fractions from the remaining membrane fractions. Preparation from either cell lysate or membrane fraction gives well-ordered hMPGES1 2D crystals [18]. Furthermore, sucrose or cesium chloride in a density gradient can isolate the functional target protein from its aggregated form.

The choice of detergent to solubilize a membrane protein from its lipid membrane may be tricky, since it affects the structure, stabilization, and function of the target protein. Nonionic detergents, such as n-dodecyl *β*-maltoside (DDM), n-decyl *β*-maltoside (DM), and n-octyl *β*-D-glucopyranoside (OG), are commonly used to solubilize membrane proteins. Triton X-100 works well for MAPEG members (hMPGES1: [18], rMGST1 (microsomal glutathione S-transferase 1 from* Rattus norvegicus*): [26], and hLTC_4_S: [25, 27]). Since purification is the step shared by 2D and 3D crystallization and the general principle in these two cases is also similar, some 3D crystallization examples are demonstrated together with the ones in 2D to illustrate the effect of different purification procedures. Exchange of different kinds of detergents (such as in prokaryotic inwardly rectifying potassium channels KirBac1.1 [28] and KirBac3.1 [29]) or mixture of them (such as in Kv1.2 (a voltage gated potassium channel from* Rattus norvegicus*) [30]) in the purification procedure results in successful crystallization. In some cases (such as in hLTC_4_S [25]), detergents may also mimic the hydrophobic substrate for the membrane protein. On the other hand, adding lipids in purification together with the detergents may be necessary as in Kv1.2 [30]. Although a pure sample is desired for crystallization, the protein may lose its activity following a too extensive purification, since this procedure may remove some lipids that help to maintain the integrity of membrane proteins (such as in hFLAP (5-lipoxygenase activating protein from* Homo sapiens*, a MAPEG member) [31]).

### 2.3. 2D Crystallization

When a reasonable pure sample is obtained, crystallization trials can be started. Formation of 2D crystals is due to a net entropy gain, occurring when membrane proteins are switched from the environment surrounded by detergents to lipids [32].

#### 2.3.1. 2D Crystallization Procedure

**Figure 2:**
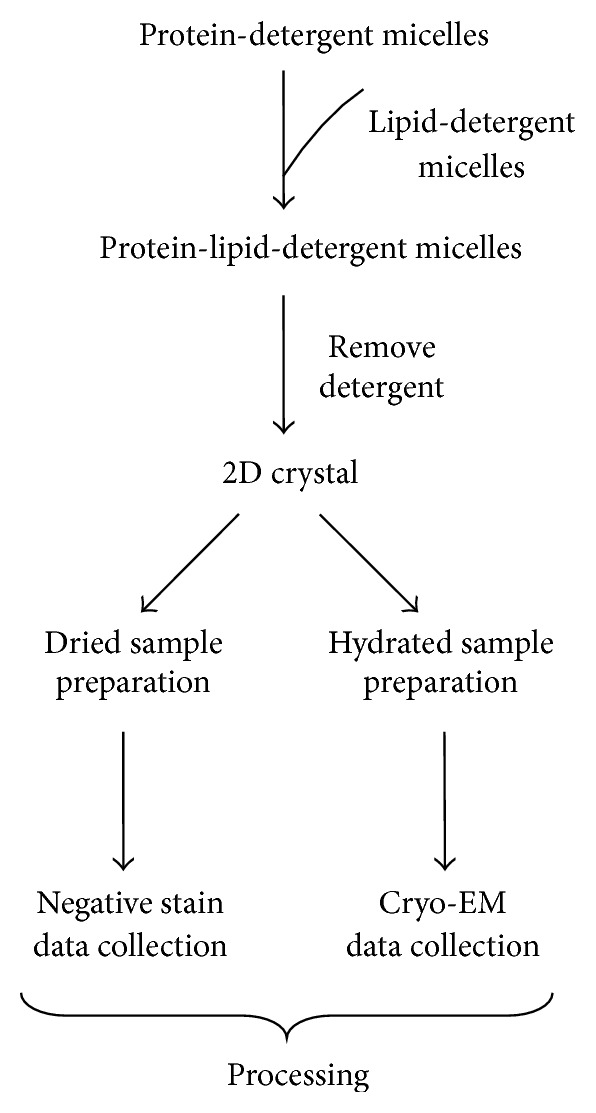
Flowchart of 2D crystallization and its following-up procedures. The key parameters in crystallization are protein, detergent, lipid, lipid-to-protein ratio, pH, buffer, additive, temperature, and detergent removal method.

2D crystallization is straightforward: the target protein with its surrounding detergents after purification is mixed with the lipid-detergent micelles to form the triple component micelles containing protein, lipid, and detergent. 2D crystals form after removal of detergent [32] (Figure 2). Dialysis, dilution, hydrophobic adsorption, and lipid monolayer are common methods for removing the detergent [33, 34]. Among them, dialysis is most widely used and works well for many proteins [33–35].

2D crystallization can be roughly divided into three stages, depending on the time when the lipid bilayer forms, when the target protein inserts into the lipid bilayer, and when the crystal contacts are established. Most 2D crystallization procedures can be explained by a two-stage mechanism, where the lipid bilayer formation and protein insertion happen simultaneously followed by establishing the crystal contacts [32, 34].

2D crystallization can result in different crystal types. Four different kinds of crystals are common: single layer sheets, stacked sheets, tubular, and vesicular types [33, 34]. Single layer sheets are desired but might be difficult to obtain. Stacked sheets are made of several layers of single sheets. Tubular or vesicular crystals give two crystalline lattices when the crystal collapses on the grid [33].

#### 2.3.2. Parameters for 2D Crystallization

Many extensive reviews describing how different parameters affect 2D crystallization have been published. One of them describes extensively the steps to obtain large and well-ordered 2D crystal of KirBac3.1 [36]. The typical parameters include protein, detergent, lipid, lipid-to-protein ratio, pH, buffer, additive, temperature, and detergent removal method [33, 35, 37]. In general, all of these parameters are critical for obtaining good crystals, although their importance does vary between different proteins. Several less discussed points are emphasized here.

*(A) Detergents in 2D Crystallization*. Purification is the step prior to crystallization. Similar to the 3D crystallization, the choice of detergents affects the crystal quality. In addition, the presence of detergents influences the initial lipid bilayer formation and protein insertion in 2D crystallization. However, the effect of detergents for 2D crystallization is unpredictable [35]. Thus, it is advisable to set up crystallization trials from the proteins purified in various kinds of detergents. DDM, DM, OG, and Triton X-100 have been widely used to purify the target protein.

*(B) Crystallization Procedure*. Formation of triple component micelles containing protein, lipid, and detergent is obviously critical for a successful crystallization. Although in practice it is achieved by just mixing the protein-detergent micelles with the lipid-detergent micelles, followed by incubation for a certain period of time and then setting up dialysis, many reactions are carried out during this period. Unfortunately, a lot remains to be investigated and it is possible that these reactions may vary for the same protein when it is surrounded by different kinds of detergents. Various procedures can be tested, for example, mixture of different kinds of lipids (such as in KirBac3.1 [36]) or detergents (such as in Kv1.2 [38]); incubation of the triple component micelles at 4°C (such as in KirBac3.1 [29]) or room temperature (such as in hMPGES1 [18]); and dialysis in a shifted temperature profile (such as in MlotiK1 (a non-voltage gated potassium channel from* Mesorhizobium loti*) [39]) or at a constant temperature (such as in hMPGES1 [18]). The resulting crystals of Kch (a ligand gated potassium channel from* E. coli*) [40, 41] and rMGST1 do have different diffracting order with different crystallization procedures.

**Figure 3:**
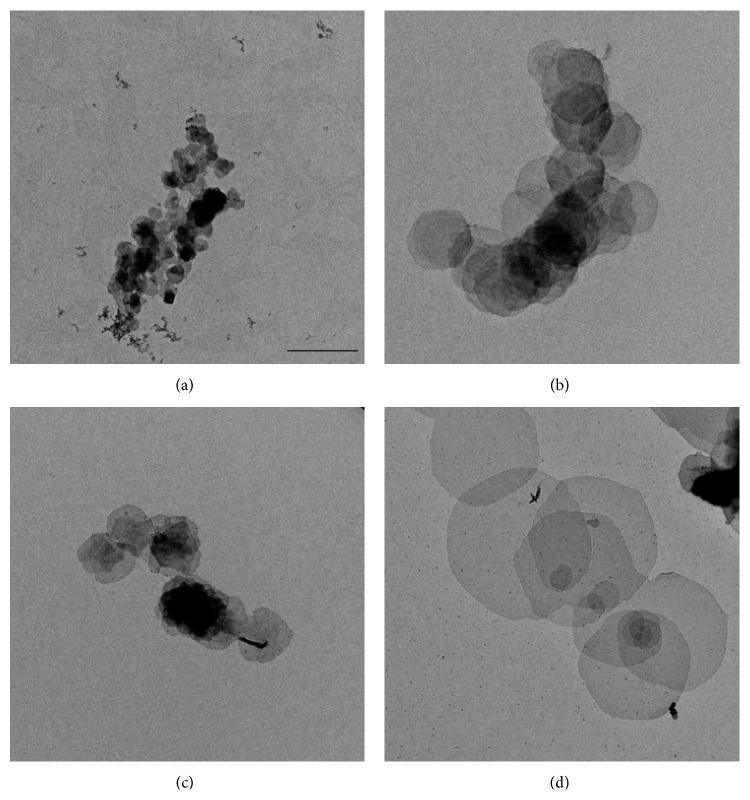
Negatively stained rMGST1 crystals. The effects of different salt concentrations and dialysis temperatures are shown in the figure. (a) Previous condition [37]. (b) With additional CaCl_2_. (c) With an increased dialysis temperature. (d) Combined effect of salt and higher temperature. The size of the crystals improves significantly in (d) as compared to (a–c). A number of single layer sheets were obtained in (d), making data collection possible. The crystals in these four conditions were embedded in 1% uranyl acetate. These four images were taken at the same nominal magnification of 5000x and the scale bar is 2 *μ*m.

**Figure 4:**
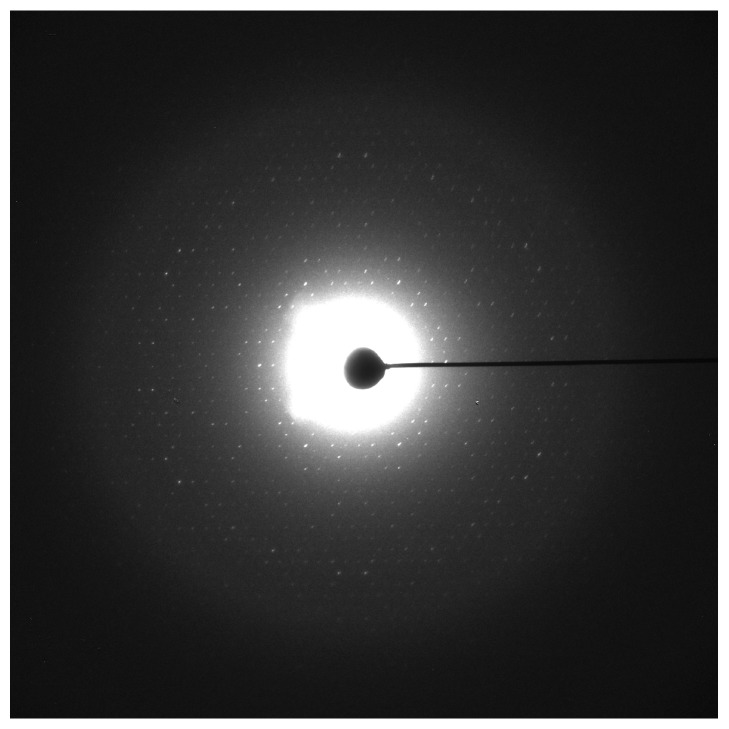
Electron diffraction pattern of rMGST1. rMGST1 crystals were grown using the condition in Figure 3(d) and embedded in trehalose. The data shown here was collected from an untilted crystal. The unit cell parameters of the p6 symmetry plane group are *a* = *b* = 81.8 Å, *γ* = 60°. The crystals grown using the current condition are isomorphic to those obtained earlier [52], which were used to calculate the projection structures [53] and the 3D reconstruction [52].

*(C) All Parameters Work Together*. A successful condition resulting in good quality crystals is quite often a combination of the parameters listed above. For instance, crystals of recombinantly overexpressed rMGST1 were small using the previous crystallization parameters [37] (Figure 3(a)). Adding CaCl_2_ and increasing the dialysis temperature to 30°C at the same time increased the size and quality of the crystals (Figure 3(d)). However, CaCl_2_ (Figure 3(b)) or 30°C (Figure 3(c)) alone does not have such an effect. At liquid nitrogen temperature the large and well-ordered crystals diffract to a resolution of 3 Å (Figure 4). Automated crystallizations with 96-well plates [35, 42] and robotic screening of the crystallization results can speed up this step [43–45].

*(D) Repetition*. Many steps in the electron microscopic studies need to be repeated and be as reproducible as possible. It is essential that the protein samples for screening the crystallization conditions are identical, which is especially critical at the beginning of the project. After a proper condition is found, collecting the whole data set also requires identical crystals. Since usually one crystal only tolerates one exposure, many image/diffraction patterns from isomorphous crystals need to be merged together. Nowadays, all these steps can be automated to improve the speed and success of each project [35, 42, 43, 45–47].

Several recommendations are listed below to increase the success rate. Firstly, set up crystallizations in parallel and have several trials each time. Secondly, beware of the decay of the protein samples. The activity of protein may decrease with time. Thirdly, only change one parameter at a time and systemically alter other parameters when optimizing the crystallization condition. Fourthly, use newly prepared materials. For example, glutathione is made freshly for MAPEG members in each crystallization trial, since the reduced glutathione oxidizes with time.

### 2.4. Sample Preparation

Biological samples must be preserved either frozen or dried in order to avoid evaporation of water in the electron microscope. The samples can be embedded in heavy metal salts and dried (called negative staining) or embedded in vitrified ice or medium by rapidly freezing in a low temperature (called cryo). In this section, these preparation methods are introduced one by one followed by a discussion of another important component, the grid, on which the sample is loaded.

#### 2.4.1. Negative Staining

Negative staining is mainly applied to screen the quality of the sample, for example, the oligomeric state of the protein or formation of crystal. The image is formed by the contrast between the heavy metal signal in the background and the light element signal from the biological sample [48]. Commonly used stains are uranyl acetate, uranyl formate, ammonium molybdate, and sodium phosphotungstate [49]. Usually 1-2% uranyl acetate solution works (rMGST1 crystal embedded in uranyl acetate is shown in Figure 3). However, incorporation of other additives, for example, trehalose [50], may be beneficial. Washing with water may be necessary in some cases to reduce the high salt and/or detergent content in the sample buffer [48, 51].

Although heavy metals provide great contrast, the negative staining displays only the contour of the protein molecules. Thus, the internal molecular detail is invisible and the obtained information is limited to 12–15 Å resolution after image processing [54]. The protein samples may be distorted by the stain as well [49, 55]. Images may appear differently when different kinds of stain are applied, which reflects the potential interaction between the stain and the protein sample [51].

#### 2.4.2. Vitrified Ice Embedding Cryo-EM

Since radiation damage can be reduced in cryo-temperatures [56] and the preserved samples can maintain their close to native structures, the data sets for structural determination are collected mainly from cryo-samples, if possible.

The protein samples can be frozen by directly plunging into liquid ethane cooled by liquid nitrogen. This process should be fast enough to allow vitrification of ice instead of formation of ice crystals. Liquid ethane is commonly used due to its high cooling efficiency [57], although other possible cryogens also exist. Since the contrast in cryo-sample images is from the scattering differences between the ice, protein, and lipid/detergent, the protein sample may be invisible if the molecular weight of the protein is low [48]. In these cases, cryo-negative staining with, for example, ammonium molybdate may solve the problem [49, 58].

In cryo-specimens, the ice thickness should be proper. If the ice layer is too thin, the sample will be dried or may give artefacts (such as specimen flattening); on the other hand, if the ice layer is too thick, the image quality is decreased [59].

Vitrified ice embedding is widely used for single particle molecules [48]. Different kinds of grid and/or stain may facilitate discerning the detergent solubilized samples in the micrograph [49, 60].

#### 2.4.3. Sugar Embedding Cryo-EM

Besides vitrified ice, the sample can be embedded in other preserving media, for example, glucose, tannin, or trehalose, which mimic the effect of water by hydrogen bonding to the sample [59]. Sugar embedding is widely used for 2D crystals (see also Figure 4), although direct plunge-freezing without any additive may work as well [61–63].

#### 2.4.4. Grid Handling

Either 2D crystals or single protein molecules are deposited on the grid, which is then inserted into electron microscope.

Glow discharging the grid to make the film more hydrophilic is a common routine for sample preparation and its efficiency is mainly depending on the property of the sample and how it is performed [59, 64]. Glow discharging is probably a critical step for single protein molecules to adsorb to the grid in SPR, whereas its usage for 2D crystals in EC may be a parameter to be tested for each project [48, 65].

Holey carbon grids are used for SPR, where images showing different orientations of single particles in the holes are recorded. Some protein molecules have a tendency to locate to the edges of the holes, a problem that becomes severe when the ice layer is becoming thinner. Besides a suitable ice thickness and the glow discharge treatment mentioned above, a thin continuous carbon layer can alleviate this problem. Carbon film can reduce the beam-induced charging and movement of the specimen in the electron microscope and it is commonly used as a support for 2D crystals [48].

The carbon film flatness is crucial for 2D crystal data collection, particular for images tilted at high angles [59]. It can be achieved by choosing a high quality carbon source, preevaporating prior to actual carbon layer preparation, and by using a molybdenum grid, which prevents wrinkling of 2D crystals at cryo-temperatures [59, 66, 67].

The back-injection method helps to preserve the high resolution information and is most frequently used for preparing 2D crystal specimens [68]. To increase specimen flatness on the grid, a second carbon layer can be deposited to the side where the sample is exposed to air [69, 70]. This carbon sandwich method can reduce the charging effect as well [48, 59].

### 2.5. Processing of 2D Crystal Images

Collected images and/or diffraction patterns of 2D crystals are further computationally processed by different programs (such as MRC [71], 2dx [72, 73], and IPLT [74]) to construct a 3D volume of the object. The theory and image processing procedures are discussed elsewhere [66, 75–77] and not reviewed here.

The EC method was first developed based on the studies of bacteriorhodopsin [78], in which large and well-ordered 2D crystals were processed. However, in most projects it would be difficult and time-consuming to search for a proper condition to obtain the crystals in equal quality as in bacteriorhodopsin. In addition, crystals are not perfect if the unit cells are slightly displaced with respect to each other (a property called mosaicity). Although the crystalline area can be boxed and processed to extract the structural information, the result is not accurate if the crystal is small and deviates from an ideal one. The “unbending” step in a standard EC procedure can correct the translationally distorted unit cells [71, 79]. However, this procedure does not work well for rotational variation or large translational errors of unit cells. Although SPR is aimed for single particles, even a crystal nucleus having several unit cells can be treated as “single particles” for SPR. Indeed, during recent years it has been shown that SPR can potentially correct for local variations that are not taken into account by EC. Therefore, the SPR method can be used for analyzing 2D crystal data as well [80–82]. This newer approach takes advantage of both EC and SPR and is suitable for small and locally disordered 2D crystals.

The hitherto highest resolution obtained with biological samples by EC is the structure of the water pore aquaporin-0, which at 1.9 Å resolution revealed lipid-protein interactions [83]. The potential of modern SPR in structural determination of membrane proteins has become evident with the solved structures of ion channels TRPV1 and TRPA1 at 3.4 Å [84] and 4.2 Å [85], ryanodine receptor at 3.8 Å [86], and *γ*-secretase at 4.3 Å [87] resolutions without crystallization.

## 3. Outlook

From a modest beginning, electron microscopy has emerged as a powerful tool in membrane protein structural determination. Automation of screening of 2D crystallization trials as well as the data acquisition step [35, 42–47], recent introduction of direct electron detectors [88], and continuous development in image processing programs have both speeded up the whole process and improved data quality. Introducing new platforms like reconstituting membrane proteins in liposomes [89], nanodiscs [90], or amphipols [91] or producing membrane protein-enriched extracellular vesicles [92] is other means that can boost future structural studies of these delicate but important proteins.
